# Supplementary material for: Ultrafast hot-carrier dynamics in ultrathin monocrystalline gold
Source: Nat Commun. 2024 Jan 24;15:703. doi: 10.1038/s41467-024-44769-3 (PMC10808103; doi:10.1038/s41467-024-44769-3)
Supplement: Supplementary file 1 — Supplementary Information [file 41467_2024_44769_MOESM1_ESM.pdf]

Supplementary Information

Ultrafast Hot-Carrier Dynamics in Ultrathin Monocrystalline Gold

Can O. Karaman*[1], Anton Yu. Bykov†[2], Fatemeh Kiani[1], Giulia Tagliabue‡[1] and Anatoly V. Zayats§[2]

[1]Laboratory of Nanoscience for Energy Technologies (LNET), STI, École Polytechnique Fédérale de Lausanne, 1015 Lausanne, Switzerland
[2]Department of Physics and London Centre for Nanotechnology, King's College London, London WC2R 2LS, UK

Supplementary Note 1: Transient reflectivity simulations

The time dependence of the optical properties of metals can be described considering two coupled thermal systems made up of the electron and the phonon baths when electron heating, resulting in a nonequlibrium electron distribution, is introduced [1, 2]. A phenomenological description of the evolution of the electron gas can be obtained considering the nonthermalized electrons, created by the absorbed excitation pulse, which act as a heat reservoir and decay by exchanging energy through electron-electron scattering with the colder electrons until the electron system reaches thermal equilibrium. Electron-phonon scattering then equilibrates the temperatures of the electron and lattice subsystems. By taking into account the energy content of the electron gas, the excitation-induced evolution of the coupled electron-phonon system can be described by three coupled differential equations as [2]

$$\frac{\partial N(t)}{\partial t} = -\overline{\gamma_e} N(t) - \frac{G}{C_e} N(t) + P_{\mathrm{abs}}(t) \tag{S1}$$

$$C_e \frac{\partial T_e(t)}{\partial t} = -G(T_e(t) - T_l(t)) + \overline{\gamma_e} N(t) \tag{S2}$$

$$C_l \frac{\partial T_l(t)}{\partial t} = G(T_e(t) - T_l(t)) + \frac{G}{C_e} N(t) \tag{S3}$$

where C_e and C_l are the electron and lattice heat capacities, respectively, T_e and T_l are the electron and lattice temperatures, respectively, G is the electron-phonon coupling constant, N is the energy density stored in the nonthermalized electrons, $\overline{\gamma_e}$ is the average scattering rate of the nonthermalized electrons, which is obtained by averaging $\tau_{ee}^{-1}(\varepsilon) = \gamma_{ee}(\varepsilon) = (D_e/\hbar)(\varepsilon)^2$ with ε and D_e being the excited electron energy measured from E_F and the proportionality constant extracted from *ab initio* calculations of electron lifetimes [3, 4], respectively. P_{abs} is

*Equal contributions
†Equal contributions
‡Correspondence email address: giulia.tagliabue@epfl.ch
§Correspondence email address: a.zayats@kcl.ac.uk

the absorbed pump-pulse power in the unit volume of the metal, which depends on a pump fluence F as

$$P_{\text{abs}}(t) = \sqrt{\frac{2}{\pi}}\frac{A(\lambda)F}{L\tau_p}\exp(-2t^2/\tau_p^2) \tag{S4}$$

where $A(\lambda)$ is the metal absorbance at the excitation wavelength, L is the thickness of the metal, τ_p is the half-width of the pump pulse at the $1/e^2$ power. The corresponding change in the thermalized and nonthermalized electron occupancy induced by the optical excitation is given by [5–7]

$$\Delta f_T(\varepsilon, t) = f_0(\varepsilon, T_e(t)) - f_0(\varepsilon, T_0) \tag{S5}$$

$$\Delta f_{NT}(\varepsilon, t) = \frac{1}{C}\cdot\Delta_{NT}(\varepsilon)\int_{-\infty}^{t} P_a(t')e^{-(t-t')/\tau(\varepsilon)}dt' \tag{S6}$$

where f_0 is the Fermi-Dirac distribution and $\Delta_{NT}(\epsilon) = f_0(\varepsilon - \hbar\omega_{\text{pump}})[1 - f_0(\varepsilon)] - f_0(\varepsilon)[1 - f_0(\varepsilon + \hbar\omega_{pump})]$. The normalization constant can be found from the energy conservation law as [6]

$$C = \int \Delta_{NT}(E)\text{DOS}(E)EdE \tag{S7}$$

where $\text{DOS}(E)$ is the energy density states of Au.

The excitation-induced change in the occupancy of thermalized and nonthermalized electron states causes the modulation of the interband transition in Au as $\Delta\epsilon_T(\hbar\omega, t)$ and $\Delta\epsilon_{NT}(\hbar\omega, t)$, respectively (Eq. 1 in the main text). The imaginary part of these quantities can be computed under the constant matrix element approximation in the parabolic band approximation as [5, 7–10]

$$\text{Im}[\Delta\epsilon_{T(NT)}(\hbar\omega, t)] = \frac{A_X J_{XT(NT)}(\hbar\omega, t) + A_{L_4^+}J_{L_4^+T(NT)}(\hbar\omega, t) + A_{L_{5+6}^+}J_{L_{5+6}^+T(NT)}(\hbar\omega, t)}{(\hbar\omega)^2} \tag{S8}$$

where

$$J_{iT(NT)}(\hbar\omega, t) = \int_{E_{min}}^{E_{max}} D_i(E, \hbar\omega)\Delta f_{T(NT)}(E, t)dE \tag{S9}$$

is the joint density of states (JDOS) with $D_i(E, \hbar\omega)$ being the energy distribution of the joint density of states (EDJDOS), and A_i is the square of the momentum operator matrix element which describes the transition strength for the corresponding transitions with $i = X, L_4^+, L_{5+6}^+$. The reduced masses of the electrons in the bands, and the energy separations between the bands at high symmetry points (Table S1) are taken from Refs. [9, 10]. The integration limits in Eq. S9 are calculated as in Ref. [10–12]. To fit the measured permittivity of Au for polycrystalline [13] and monocrystalline [14] gold, we set A_i for $i = X, L_4^+, L_{5+6}^+$ as fitting parameters. For PC Au, we find $A_X/A_{L_{5+6}^+} = 0.065$ and $A_X/A_{L_4^+} = 0.129$, and for MC Au, $A_X/A_{L_{5+6}^+} = 0.07$ and $A_X/A_{L_4^+} = 0.15$. The third term in Eq. (1) in the main text, related to the induced changes in the intraband permittivity, is calculated through the Drude-Sommerfeld model [15]. The intraband part of the permittivity is described as

$$\epsilon_{intra} = \epsilon_{inf} - \frac{\omega_p^2}{\omega(\omega + i\Gamma(\hbar\omega, T_e, T_l))} \tag{S10}$$

where $\omega_p = 9.02$ eV is the plasma frequency of Au and $\epsilon_{inf} = 3$ was set to fit the PC Au permittivity [13, 16]. Pump-induced modulation of the Drude damping parameter, $\Gamma(\hbar\omega, T_e, T_l)$, is [17, 18]

$$\Gamma(\hbar\omega, T_e, T_l) = \Gamma_{e-e}(\hbar\omega, T_e) + \Gamma_{e-ph}(T_l) \tag{S11}$$

Table S1: Band-structure parameters near the X- and L-symmetry points used in the model for PC and MC Au.

	Mass (m_0)										Energy (eV)				
	$m_{p\parallel}^X$	$m_{p\perp}^X$	$m_{d\parallel}^X$	$m_{d\perp}^X$	$m_{p\parallel}^L$	$m_{p\perp}^L$	$m_{d\parallel}^{L_4^+}$	$m_{d\perp}^{L_4^+}$	$m_{d\parallel}^{L_{5+6}^+}$	$m_{d\perp}^{L_{5+6}^+}$	$\hbar\omega_7^X$	$\hbar\omega_6^X$	$\hbar\omega_{4+}^L$	$\hbar\omega_{4-}^L$	$\hbar\omega_{5+6}^L$
PC	0.12	0.22	0.91	0.75	0.25	0.23	0.57	0.49	0.7	0.63	1.72	1.72	2.6	0.65	1.45
MC	0.2	0.22	0.9	0.8	0.15	0.13	0.57	0.49	0.95	0.63	1.65	1.72	2.6	0.65	1.55

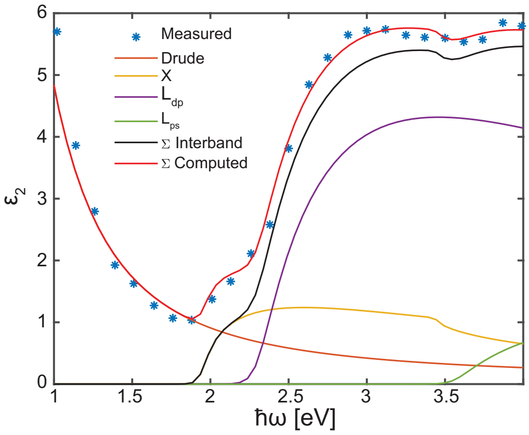

Supplementary Fig. 1: The imaginary part of (blue stars) the experimental data for the permittivity of PC Au [13] and (red curve) the permittivity calculated from the model. The contributions of the different electron transitions to the permittivity are also shown.

with

$$\Gamma_{e-e}(\hbar\omega, T_e) = \frac{\pi^3}{12}\beta\Delta(1/\hbar E_F)[(k_B T_e)^2 + (\hbar\omega)^2] \tag{S12}$$

and

$$\Gamma_{e-ph}(T_l) = \frac{1}{\tau_0}\left[\frac{2}{5} + 4\left(\frac{T_l}{\Theta}\right)^5 \int_0^{\Theta/T_l} \frac{z^4}{e^z - 1} dz\right] \tag{S13}$$

where E_F= 5.55 eV is the Fermi energy of Au, $\beta \approx 0.55$ for noble metals is the average electron scattering probability over the Fermi surface, $\Delta \approx 0.75$ is the fractional Umpklapp scattering [19], $\Theta = 170$ K is the Debye temperature for Au, and a $\tau_0^{-1} \approx = 6.77$ THz for Au is obtained from fitting the Au bulk permittivity for frequencies below 2.5 eV, which is the interband transition onset in Au [20].

The calculated as described spectrum of the imaginary part of a Au permittivity ϵ_2 fits well the experimental data and allow to separate the contributions from different electron processes to the permittivity (Supplementary

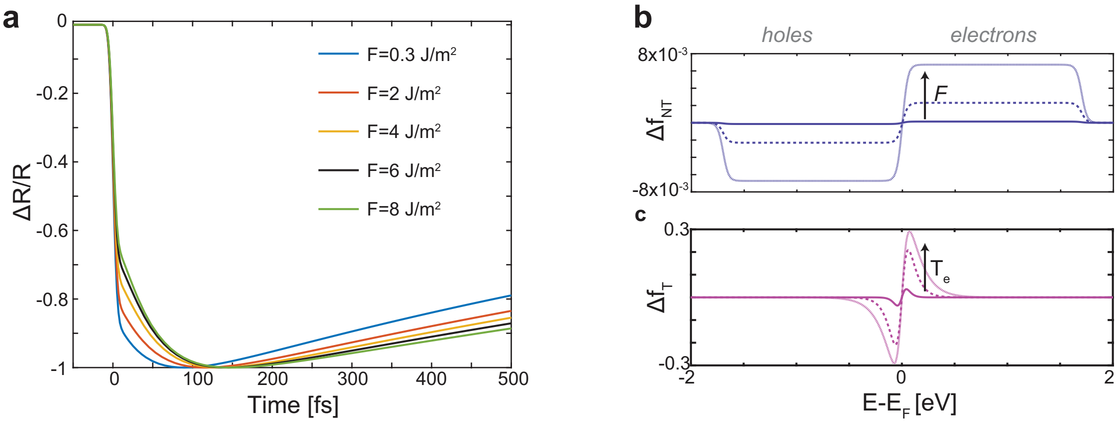

Supplementary Fig. 2: **a** Simulated dynamics of normalised $\Delta R/R$ at different excitation fluences. **b,c** The electron energy spectra of the instantaneous changes in (b) the nonthermal electron distribution, Δf_{NT} at $F = 0.2\ \ \mathrm{Jm}^{-2}$ (solid line) ,$F = 5\ \ \mathrm{Jm}^{-2}$ (dashed line), and $F = 11.5\ \ \mathrm{Jm}^{-2}$ (dotted line), and (c) the corresponding thermalised electron distribution, Δf_{NT}, for maximum $\Delta T_e = 50$ K (solid line), 500 K (dashed line) and 1000 K (dotted line). The excitation wavelength corresponds to 1.7 eV.

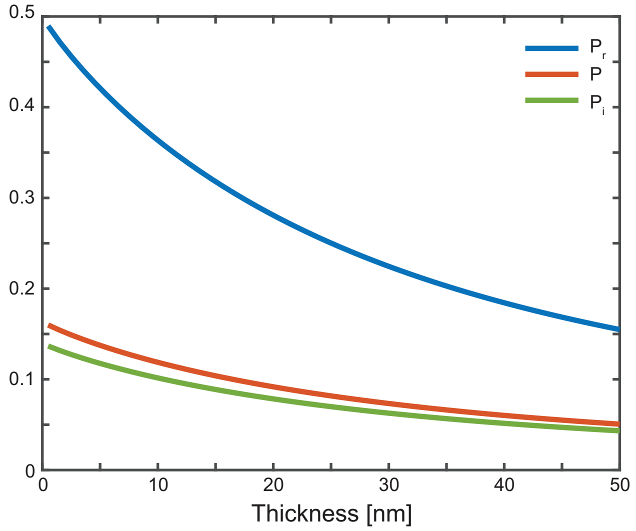

Supplementary Fig. 3: The thickness dependence of P, P_i and P_r for a Au slab on a semiconductor substrate for the excitation at $\hbar\omega = 1.7$ eV and a barrier $\phi_b = 1$ eV.

Fig. 1). The corresponding real part of permittivity is calculated using the Kramers-Kronig analysis. The transfer matrix method [21] is then used to simulate $\Delta R(t)/R$ using $\Delta\epsilon(\hbar\omega, t)$ for required excitation fluences (Supplementary Fig. 2a). The same procedure is applied to simulate the permittivity and reflectivity changes for MC Au using the experimental data from Ref. [14].

Supplementary Note 2: Hot-electron transfer efficiency simulation

The Fowler's law is widely used to describe photoemission from an emitter to a collector in the case of bulk materials but needs to be corrected for thin films and nanostructures. The Fowler's law only accounts for a single interaction with the metal-semiconductor interface and disregards the carrier interactions. However, when the thickness is smaller than a hot-electron mean-free path, the quasi-elastic scattering between electrons and phonons can efficiently randomize the momentum of the hot electrons and increase the transfer efficiency by increasing the probability of the momentum matching. Therefore, we assume complete relaxation of the momentum conservation during the hot-electron transfer in our case. The probability that an electron is excited to particular energy, $E = E_F + \varepsilon$, is given by the product of the electron density of states, DOS(ε), at the initial and final electron energies, normalized by the same product integrated over all possible initial and final electron energy combinations [22]:

$$P_\varepsilon(\varepsilon) = \frac{\mathrm{DOS}(\varepsilon + E_F)\mathrm{DOS}(\varepsilon + E_F - \hbar\omega)}{\int_{E_F}^{E_F+\hbar\omega} \mathrm{DOS}(E')\mathrm{DOS}(E' - \hbar\omega)dE'} \tag{S14}$$

where $\hbar\omega$ is the excitation photon energy. We assume the parabolic band approximation for the intraband transitions, so that $DOS(\varepsilon) \propto \sqrt{\varepsilon}$. We also assume that electron-phonon scattering causes a minimal loss of hot-electron energy, and the main energy loss takes place through electron-electron scattering. In this case, the probability that a hot electron will reach the interface is determined by the mean free path, λ_e:

$$P_r(L, \varepsilon) = \frac{1}{2\pi L}\int_0^L \int_{-\pi/2}^{\pi/2} \exp\big(-\frac{z}{\cos\theta\lambda_e(\varepsilon)} - \frac{z}{\lambda_p}\big)dzd\theta \tag{S15}$$

where L is the thickness of a Au film, θ is the angle between the normal to the interface and the direction of electron propagation, λ_p is the penetration depth of the incoming photon, and z is the normal distance from the

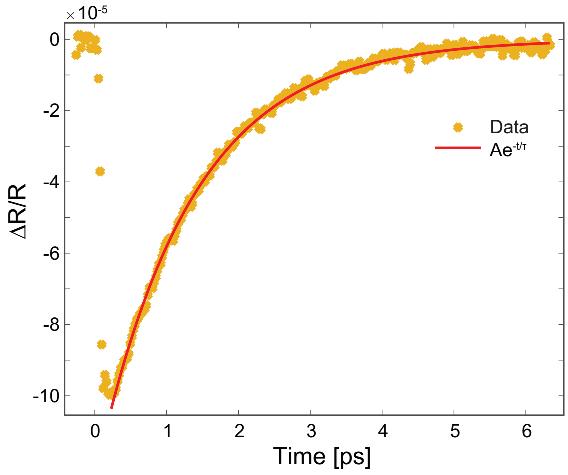

Supplementary Fig. 4: The measured $\Delta R/R$ for a 15-nm-thick Au MC flake (yellow) and the best fit single exponential dependence (red).

interface. The factors of the $1/L$ and $1/(2\pi)$ come from assuming an equal number of electrons in all dz slices and isotropic momentum distribution, respectively. The total probability $P(\hbar\omega)$ for an excited electron generated by an incident photon of energy $\hbar\omega$ to be injected into a semiconductor is

$$P(\hbar\omega) = \int_{\phi_b}^{\hbar\omega} P_\varepsilon(\varepsilon) P_r(L,\varepsilon) d\varepsilon \tag{S16}$$

where ϕ_b is a barrier height. The fraction of the absorbed energy injected into the semiconductor by the transferred hot electrons, P_i, is then [23]

$$P_i(\hbar\omega) = \int_{\phi_b}^{\hbar\omega} \frac{\varepsilon}{\hbar\omega} P_\varepsilon(\varepsilon) P_r(L,\varepsilon) d\varepsilon \tag{S17}$$

The simulations show that as the thickness of a Au slab decreases, P, P_i, and P_r increase. Therefore, with a higher probability of injection of hot electrons, a higher amount of absorbed energy is transferred to the semiconductor (Supplementary Fig. 3).

Supplementary Note 3: Electron-Phonon relaxation lifetime analysis

The pump and probe pulse length in the experiments is 8 fs which is much smaller than the analysed relaxation times ($\tau_{rise} \approx 250$ fs and $\tau_{e-ph} \approx 2$ ps). Deconconvolution of the measured $\Delta R/R$ with the 8-fs-long Gaussian shaped pulse, did not show any changes in the obtained lifetimes since the laser pulses are much shorter compared to the changes in the reflectivity. As an example, Supplementary Fig. 4 shows the exponential fit to find τ_{e-ph}. A single exponential function is enough to determine τ_{e-ph} considering it as the fitting parameter. The single exponential is fitted from the absolute maximum of $\Delta R/R$. In this specific example, $\tau_{e-ph} \approx 1.27$ ps was obtained.

Supplementary Note 4: Sample characterisation and optical set-up

Based on the AFM images of the PC Au thin film and MC Au flake (Supplementary Fig. 5), the root mean squared surface roughness is approximately 1.5 nm and 200 pm, respectively. For this roughness level, we do not expect to observe any significant scattering from either film in the visible and near-infrared spectral ranges. Therefore,

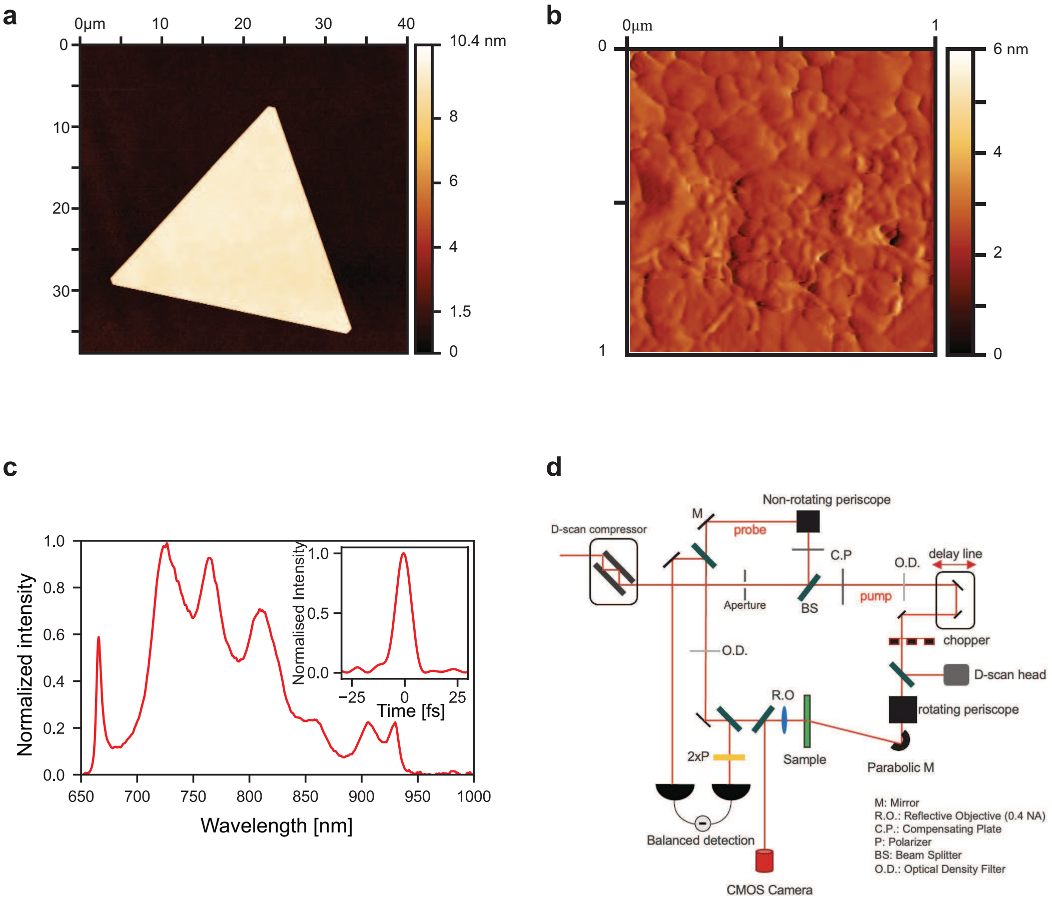

Supplementary Fig. 5: **a,b** AFM images of (a) MC Au flake and (b) polycrystalline Au thin film used in this study. **c** Spectrum of a 8 fs laser pulse used in the pump-probe measurements. Inset shows the length of a pulse. **d** Schematics for the degenerate pump-probe measurements.

the simulated reflection, absorption, and transmission spectra (Fig. 1c) fully represent the optical properties of the studied Au thin films.

In the experiments, additional light scattering may arise due to the microflake boundaries if the beam is larger than a studied microflake. In our case, the pump and probe beam which is about 10 μm and 1 μm in diameter, respectively, are always centered on the flake, which has the dimensions of 30×30 μm^2. Therefore, we do not observe any scattering from the edges of the MF in the experiments. For the PC Au film, the average grain size is 50 nm (Supplementary Fig. 5b) and the pump beam interacts with multiple grains, so that the scattering of the excited electrons on the grain boundaries is important.

References

1. Schoenlein, R. W., Lin, W. Z., Fujimoto, J. G. & Eesley, G. L. Femtosecond studies of nonequilibrium electronic processes in metals. *Phys. Rev. Lett.* **58,** 1680–1683 (1987).
2. Sun, C.-K., Vallée, F., Acioli, L. H., Ippen, E. P. & Fujimoto, J. G. Femtosecond-tunable measurement of electron thermalization in gold. *Phys. Rev. B* **50,** 15337–15348 (1994).
3. Brown, A. M., Sundararaman, R., Narang, P., Schwartzberg, A. M., Goddard, W. A. & Atwater, H. A. Experimental and ab initio ultrafast carrier dynamics in plasmonic nanoparticles. *Phys. Rev. Lett.* **118,** 087401 (2017).

4. Brown, A. M., Sundararaman, R., Narang, P., Goddard, W. A. & Atwater, H. A. Ab initio phonon coupling and optical response of hot electrons in plasmonic metals. *Phys. Rev. B* **94,** 075120 (2016).

5. Zavelani-Rossi, M., Polli, D., Kochtcheev, S., Baudrion, A.-L., Béal, J., Kumar, V., Molotokaite, E., Marangoni, M., Longhi, S., Cerullo, G., Adam, P.-M. & Della Valle, G. Transient optical response of a single gold nanoantenna: the role of plasmon detuning. *ACS Photonics* **2,** 521–529 (2015).

6. Della Valle, G., Conforti, M., Longhi, S., Cerullo, G. & Brida, D. Real-time optical mapping of the dynamics of nonthermal electrons in thin gold films. *Phys. Rev. B* **86,** 155139 (2012).

7. Dal Conte, S., Conforti, M., Petti, D., Albisetti, E., Longhi, S., Bertacco, R., De Angelis, C., Cerullo, G. & Della Valle, G. Disentangling electrons and lattice nonlinear optical response in metal-dielectric Bragg filters. *Phys. Rev. B* **89,** 125122 (2014).

8. Stoll, T., Maioli, P., Crut, A., Del Fatti, N. & Vallée, F. Advances in femto-nano-optics: ultrafast nonlinearity of metal nanoparticles. *The European Physical Journal B* **87,** 260 (2014).

9. Zilli, A., Langbein, W. & Borri, P. Quantitative measurement of the optical cross sections of single nano-objects by correlative transmission and scattering microspectroscopy. *ACS Photonics* **6,** 2149–2160 (2019).

10. Masia, F., Langbein, W. & Borri, P. Measurement of the dynamics of plasmons inside individual gold nanoparticles using a femtosecond phase-resolved microscope. *Phys. Rev. B* **85,** 235403 (2012).

11. Guerrisi, M., Rosei, R. & Winsemius, P. Splitting of the interband absorption edge in Au. *Phys. Rev. B* **12,** 557–563 (2 July 1975).

12. Rosei, R. Temperature modulation of the optical transitions involving the Fermi surface in Ag: Theory. *Phys. Rev. B* **10,** 474–483 (2 July 1974).

13. Johnson, P. B. & Christy, R. W. Optical constants of the noble metals. *Phys. Rev. B* **6,** 4370–4379 (1972).

14. Olmon, R. L., Slovick, B., Johnson, T. W., Shelton, D., Oh, S.-H., Boreman, G. D. & Raschke, M. B. Optical dielectric function of gold. *Phys. Rev. B* **86,** 235147 (2012).

15. Patterson, J. D. & Bailey, B. C. in *Solid-State Physics: Introduction to the Theory* 649–704 (Springer International Publishing, Cham, 2018).

16. Neira, A. D., Olivier, N., Nasir, M. E., Dickson, W., Wurtz, G. A. & Zayats, A. V. Eliminating material constraints for nonlinearity with plasmonic metamaterials. *Nature Communications* **6,** 7757 (2015).

17. Alabastri, A., Tuccio, S., Giugni, A., Toma, A., Liberale, C., Das, G., Angelis, F. D., Fabrizio, E. D. & Zaccaria, R. P. Molding of plasmonic resonances in metallic nanostructures: dependence of the non-linear electric permittivity on system size and temperature. *Materials* **6,** 4879–4910 (2013).

18. Holstein, T. Theory of transport phenomena in an electron-phonon gas. *Annals of Physics* **29,** 410–535 (1964).

19. Beach, R. T. & Christy, R. W. Electron-electron scattering in the intraband optical conductivity of Cu, Ag, and Au. *Phys. Rev. B* **16,** 5277–5284 (1977).

20. Thèye, M.-L. Investigation of the optical properties of Au by means of thin semitransparent films. *Phys. Rev. B* **2,** 3060–3078 (1970).

21. Katsidis, C. C. & Siapkas, D. I. General transfer-matrix method for optical multilayer systems with coherent, partially coherent, and incoherent interference. *Appl. Opt.* **41,** 3978–3987 (2002).

22. Leenheer, A. J., Narang, P., Lewis, N. S. & Atwater, H. A. Solar energy conversion via hot electron internal photoemission in metallic nanostructures: Efficiency estimates. *Journal of Applied Physics* **115,** 134301 (2014).

23. Ratchford, D. C., Dunkelberger, A. D., Vurgaftman, I., Owrutsky, J. C. & Pehrsson, P. E. Quantification of efficient plasmonic hot-electron injection in gold nanoparticle–TiO2 films. *Nano Letters* **17,** 6047–6055 (2017).
